# Biogeography of Cryoconite Bacterial Communities Across Continents

**DOI:** 10.3390/microorganisms14010162

**Published:** 2026-01-11

**Authors:** Qianqian Ge, Zhiyuan Chen, Yeteng Xu, Wei Zhang, Guangxiu Liu, Tuo Chen, Binglin Zhang

**Affiliations:** 1 Yulong Snow Station of Cryosphere and Sustainable Development, State Key Laboratory of Cryospheric Science and Frozen Soil Engineering, Northwest Institute of Eco-Environment and Resources, Chinese Academy of Sciences, Lanzhou 730000, China; gq12204113@outlook.com (Q.G.); chenzhiyuan22@mail.ucas.ac.cn (Z.C.); xuyeteng19@mails.ucas.edu.cn (Y.X.); 2 University of Chinese Academy of Sciences, Beijing 100049, China; 3 Key Laboratory of Extreme Environmental Microbial Resources and Engineering of Gansu Province, Lanzhou 730000, China; ziaoshen@163.com (W.Z.); liugx@lzb.ac.cn (G.L.); 4 Key Laboratory of Desert and Desertiffcation, Northwest Institute of Eco-Environment and Resources, Chinese Academy of Sciences, Lanzhou 730000, China

**Keywords:** biogeography, bacterial community, glacier, cryoconite, human development index

## Abstract

The geographic distribution patterns of microorganisms and their underlying mechanisms are central topics in microbiology, crucial for understanding ecosystem functioning and predicting responses to global change. Cryoconite absorbs solar radiation to form cryoconite holes, and because it lies within these relatively deep holes, it faces limited interference from surrounding ecosystems, often being seen as a fairly enclosed environment. Moreover, it plays a dominant role in the biogeochemical cycling of key elements such as carbon and nitrogen, making it an ideal model for studying large-scale microbial biogeography. In this study, we analyzed bacterial communities in cryoconite across a transcontinental scale of glaciers to elucidate their biogeographical distribution and community assembly processes. The cryoconite bacterial communities were predominantly composed of Proteobacteria, Cyanobacteria, Bacteroidota, and Actinobacteriota, with significant differences in species composition across geographical locations. Bacterial diversity was jointly driven by geographical and anthropogenic factors: species richness exhibited a hump-shaped relationship with latitude and was significantly positively correlated with the Human Development Index (HDI). The significant positive correlation may stem from nutrient input and microbial dispersal driven by high-HDI regions’ industrial, agricultural, and human activities. Beta diversity demonstrated a distance-decay pattern along spatial gradients such as latitude and geographical distance. Analysis of community assembly mechanisms revealed that stochastic processes predominated across continents, with a notable scale dependence: as the spatial scale increased, the role of deterministic processes (heterogeneous selection) decreased, while stochastic processes (dispersal limitation) strengthened and became the dominant force. By integrating geographical, climatic, and anthropogenic factors into a unified framework, this study enhances the understanding of the spatial-scale-driven mechanisms shaping cryoconite bacterial biogeography and emphasizes the need to prioritize anthropogenic influences to predict the trajectory of cryosphere ecosystem evolution under global change.

## 1. Introduction

Glaciers cover a considerable portion of the Earth’s surface, occupying approximately 10% of the continental land area [1], and exert profound influences on sea level, climate regulation, and freshwater supply [2]. As a key component of the Earth’s cryosphere, glaciers are characterized by extreme environmental conditions such as low temperatures, oligotrophy, and high radiation, posing significant challenges to biological survival [3]. Despite these harsh conditions, glacier surfaces host abundant and active microorganisms, supporting diverse and highly adapted microbial communities [4]. Aerosols, dust, and rock debris from various sources are deposited onto glacier surfaces and redistributed by meltwater. Since these deposits contain organic matter, microorganisms colonize and proliferate at different locations on the ice [5,6].

Cryoconite is an aggregate formed through interactions between organic matter, inorganic mineral particles, and microbial communities, typically found on the surfaces of ablation zones and in cryoconite holes [7]. In the ablation area, surface depressions accumulate wind-transported particles, which, combined with extracellular polymeric substances (EPS) secreted by microorganisms such as cyanobacteria, form spherical aggregates ranging from 0.26 to 3.5 mm in diameter [8]. The spherical morphology of cryoconite is maintained by filamentous cyanobacteria encasing the outer layer, while the interior uniformly distributes inorganic and organic particles [9]. The decomposition of organic matter produces humic substances, endowing cryoconite with high light-absorbing properties [7,10]. This optical characteristic reduces surface albedo, accelerates ice melting, and directly impacts global biogeochemical cycles and energy balance [11,12]. Cryoconite hosts a highly diverse microbial community, including bacteria, fungi, algae, archaea, and protists, which drives key biogeochemical cycles of carbon and nitrogen and makes it a recognized “hotspot” for microbial activity on glacier surfaces [13,14,15]. The first metagenomic study of cryoconite revealed that bacteria dominate the microbial community, with eukaryotes (0.6%) and archaea (0.1%) present in minimal abundances [16]. Major bacterial phyla include photosynthetic Cyanobacteria, Bacteroidota and Proteobacteria involved in organic matter degradation, as well as widespread Actinobacteriota and Firmicutes [17,18].

With the widespread adoption of molecular biology techniques such as high-throughput sequencing, the biogeographic patterns of microbial communities have become a research frontier. Current explorations of microbial geographic distribution have extensively covered various ecosystems, including oceans [19], lakes [20], wetland soils [21], and glaciers [22]. Glacial environments host a diversity of habitat types, with ice, snow, and cryoconite serving as common microbial habitats. Among these, cryoconite is gaining increasing attention as an ideal model system. The relatively enclosed and simple structure of cryoconite ecosystems significantly reduces interference from complex ecological interactions and offers high overall stability, making them natural laboratories for testing macroecological theories such as island biogeography and neutral theory [23]. As a “sink” for long-range atmospheric transport, cryoconite effectively integrates mineral dust, organic matter, and microorganisms from surrounding regions, serving as an excellent medium linking glacial environments to adjacent ecosystems [24]. The diverse, ever-changing depositional sources in cryoconite, along with its dynamic ecosystem boundaries shaped by glacial movement and seasonal meltwater, collectively undermine the explanatory power of stability-based macroecological theories and heighten spatial heterogeneity [25,26].

Due to sampling difficulties, most studies on glacial microbial biogeography have been confined to single glaciers or regional scales, focusing primarily on temporal variations [27,28,29] or differences among habitat types [30,31]. In recent years, with the accumulation of data, cross-glacier and even continental-scale studies are emerging, aiming to elucidate the core mechanisms driving microbial distribution in extreme environments. Some studies have found that cryoconite bacterial communities exhibit characteristics of island biogeography, with the strength of patterns such as distance-decay of community similarity being scale-dependent [32]. Significant differences in bacterial community structure have been observed between High Arctic glaciers and ice-marginal habitats, as well as between Arctic and Antarctic glaciers, indicating that the diversity and structure of cryoconite microbial communities are influenced by spatial scale [33,34].

Among the factors influencing cryoconite bacterial community structure, geographic distance and environmental conditions have been identified as key drivers of diversity and compositional variation. However, the impact of human activities, a potentially significant driver, has often been overlooked in previous research. Deposition of nitrogen and phosphorus compounds, organic carbon particles, and even toxic pollutants from human activities onto glacier surfaces may alter the original nutrient structure and physicochemical properties of cryoconite [35,36]. Furthermore, frequent human movement and material transport can introduce exogenous microorganisms into cryoconite environments [37]. Therefore, systematically evaluating the relative contributions of anthropogenic versus natural factors is essential for accurately predicting the responses of glacial ecosystems to global change.

To systematically reveal large-scale biogeographic patterns and disentangle the relative contributions of multiple environmental drivers, meta-analysis has emerged as a powerful methodological approach. By synthesizing publicly available datasets from independent investigations, this method overcomes the spatiotemporal limitations of individual studies, providing critical support for elucidating global-scale microbial ecological patterns. Building on this foundation, our study selects cryoconite as the research subject with two objectives: (1) to elucidate transcontinental biogeographic patterns of bacterial communities in cryoconite. (2) to dissect community assembly mechanisms in cryoconite, assessing the relative contributions of stochastic vs. deterministic processes, and to examine how environmental factors shape bacterial community composition and dynamics, thereby advancing understanding of microbial community formation and maintenance across regions.

## 2. Materials and Methods

### 2.1. Data Collection

**Figure 1:**
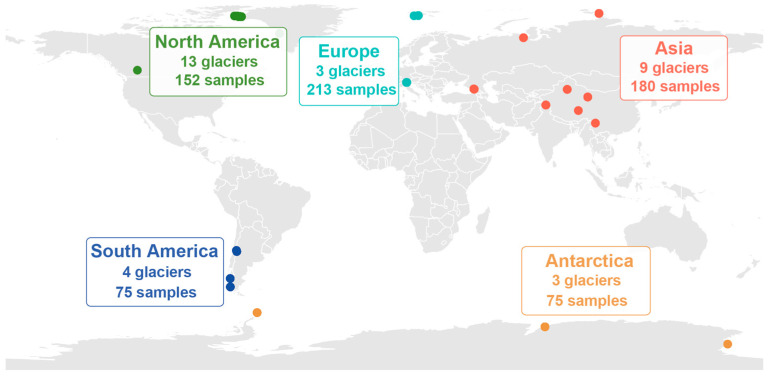
The distribution of study locations featured in the meta-analysis is depicted.

We acquired 16S ribosomal ribonucleic acid (16S rRNA) gene sequences from public databases using the search terms “cryoconite” and “16S rRNA”. The criteria for data selection were as follows: (1) only 16S rRNA sequences obtained from paired-end sequencing were collected; (2) articles must document the precise latitude and longitude of samples to facilitate geographical grouping and data analysis; (3) the 16S rRNA gene sequences must be publicly available in databases such as the National Center for Biotechnology Information (NCBI) or the European Nucleotide Archive (ENA), with corresponding accession numbers provided in the articles. A total of 695 samples from 15 papers were included in this meta-analysis [18,28,29,38,39,40,41,42,43,44,45,46,47,48,49]. Detailed information, such as accession numbers and geographic coordinates for all samples, is provided in the Supplementary Materials Table S1. These samples were grouped geographically as follows: 180 from Asia, 213 from Europe, 152 from North America, 75 from South America, and 75 from Antarctica (Figure 1). This continental classification aids in examining the differences and distribution characteristics of cryoconite bacterial communities across different continents. Continental sample size disparities may breach variance homogeneity, weaken parametric test power, bias rare species detection, and destabilize diversity indices, potentially skewing microbial community difference interpretations. Reliability can be ensured via sample standardization and non-parametric tests (e.g., Kruskal–Wallis).

To explore the drivers of cryoconite bacterial community distribution, several variables were collected at the glacier scale, including latitude (Lat), longitude (Lon), wind speed (U), mean solar radiation (MSR), mean monthly temperature (MMT), total monthly precipitation (TMP), and water vapor pressure (VP), with all climatic variables representing monthly averages for the sampling month. Additionally, global Human Development Index (HDI) data were extracted according to the countries where the glaciers are located. These variables encompass geographical location, anthropogenic factors, and climatic characteristics. As the original articles did not provide complete environmental datasets, the climatic and anthropogenic variables used herein were obtained from relevant databases. Climate factors, including maximum temperature, minimum temperature, and total precipitation, were extracted for each sampling location from WorldClim version 2 (https://www.worldclim.org/) at a spatial resolution of 30 s (~1 km^2^) [50]. The Global Human Development Index data were sourced from the UNDP (https://hdr.undp.org/data-center/), accessed on 22 September 2025. Given that UNDP has not provided HDI data for the region of Greenland, and considering its extremely low population density, this study assigns a value of 0 to the HDI of the Greenland region. All environmental variables are provided in Table S2 of the Supplementary Materials.

### 2.2. Data Processing

The collected amplicon data predominantly employ Illumina MiSeq sequencing technology, with a minority of studies utilizing Illumina HiSeq, 454 GS FLX Titanium, and Ion Torrent PGM (see Table S1 in the Supplementary Materials for detailed information). The collected 16S rRNA sequences were first subjected to quality filtering, and paired-end reads were merged using FLASH (version 1.2.11) [51]. Amplicon Sequence Variants (ASVs) were then obtained by denoising with the unoise3 algorithm in USEARCH (version 10.0.240). Subsequently, a feature table and representative sequences were generated using VSEARCH (version 2.30.0). Taxonomic annotation was performed in QIIME2 (version 2023.9) [52] with the Naive Bayes classifier [53] via the classify-sklearn algorithm, using the SILVA database (version 138) as a reference.

### 2.3. Data Analysis

A total of 727,286 raw 16S rRNA gene sequences derived from the 695 collected cryoconite samples were processed, yielding 33,675 Amplicon Sequence Variants (ASVs) after denoising. Given the disparities in sequencing coverage across samples, we normalized the data by subsampling to a uniform sequencing depth, thereby generating a standardized ASV table for downstream analyses.

To validate the effectiveness of the predefined sample groupings, Analysis of Similarities (ANOSIM) was employed to test whether between-group differences exceeded within-group variations. Alpha diversity indices (Richness and Shannon) and Bray–Curtis dissimilarities were calculated using the “vegan” package [54] in R 4.5.0. Principal Coordinate Analysis (PCoA) and Principal Component Analysis (PCA) were performed, and the significance of regional differences in community composition was assessed using Permutational Multivariate Analysis of Variance (PERMANOVA) via the adonis2 function in the “vegan” package [54]. Differences in diversity indices between groups were evaluated using the Kruskal–Wallis test, followed by Dunn’s post hoc test.

The distance-decay relationship, examining the correlation between Bray–Curtis dissimilarity and geographical distance, was analyzed by computing pairwise geographical distances between sampling sites using the “distm” function from the “geosphere” package [55].

The relationship between bacterial beta diversity and environmental factors was examined using Mantel tests in the “vegan” package [54]. Distance-based Redundancy Analysis (db-RDA) and Random Forest analysis were applied to assess the influence of environmental variables on microbial beta diversity using the “vegan” package and “randomForest” package [56]. The importance of environmental variables was ranked based on the %IncMSE (Percentage Increase in Mean Squared Error), with significance tested using the “rfPermute” package [57]. Since the environmental factor data were collected on a glacier-by-glacier basis, the analysis of the relationships between bacterial communities and environmental factors was conducted using data from 32 glaciers.

Null model analysis was conducted to quantify community assembly processes by calculating the Beta-Nearest Taxon Index (βNTI) and the Bray–Curtis-based Raup-Crick index (|RCBray|) using the picante and ape packages [58,59,60]. The ecological processes were categorized as follows: homogeneous selection (βNTI < −2), heterogeneous selection (βNTI > +2), dispersal limitation (|βNTI| < 2 and |RCBray| > +0.95), homogenizing dispersal (|βNTI| < 2 and |RCBray| < −0.95), and undominated processes/drift (|βNTI| < 2 and |RCBray| < 0.95). We then used pairwise comparisons to calculate the relative contributions of assembly processes within each scale unit (with each unit spanning 60 km) using betaNTI and RCBray values. We used linear fitting to depict the shifts in the relative contributions of various ecological assembly processes as spatial scales expanded.

## 3. Results

### 3.1. Alpha Diversity of Cryoconite Bacterial Communities

**Figure 2:**
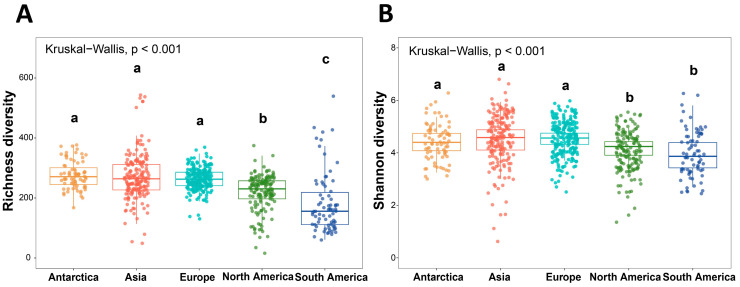
Alpha diversity of bacterial communities in cryoconite at the continental scale. (**A**) Richness diversity index of bacterial communities in cryoconite across five continents; (**B**) Shannon diversity index of bacterial communities in cryoconite across five continents. Boxes represent the upper and lower quartiles, horizontal lines indicate the median, and whiskers show 95% range. Bars sharing the same letter are not significantly different.

The alpha diversity of the cryoconite bacterial communities was assessed using the Shannon and Richness indices. The results clearly illustrate the distribution of bacterial Richness and Shannon diversity indices across cryoconite samples from different geographical regions, revealing distinct regional differences in microbial diversity (Figure 2). Regarding the Richness index, Antarctica, Asia, and Europe exhibited relatively high levels, which were significantly greater than those observed in North and South America (Figure 2A). The Shannon index, which incorporates both species richness and evenness, indicated the highest mean value in Europe (4.50 ± 0.352), followed by Asia and Antarctica, while North and South America showed the lowest mean values (Figure 2B). The differences in alpha diversity indices among regions were statistically significant (*p* < 0.001). However, post hoc tests revealed no significant differences in either the Shannon or Richness indices among Antarctica, Asia, and Europe (Table S3).

### 3.2. Taxonomic Composition of Cryoconite Bacterial Communities

Samples were grouped according to their continent of origin. The validity of this grouping was confirmed by ANOSIM, which revealed a significant difference in community structure between regions (R = 0.4948, *p* < 0.001), indicating that between-group variation substantially exceeded within-group variation (Table S4). The most pronounced dissimilarity was observed between South America and North America (R^2^ = 0.78), whereas the difference between Antarctica and Asia was the least marked (R^2^ = 0.16).

**Figure 3:**
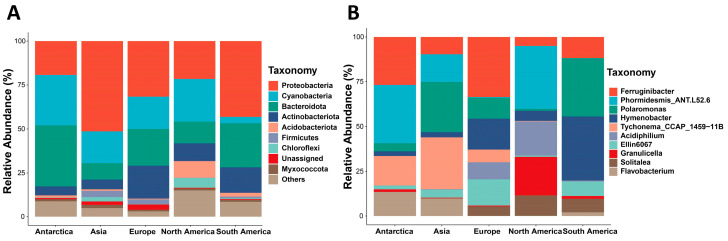
Analysis of taxonomic composition of cryoconite bacterial communities across different continents. (**A**) Phylum-level composition of cryoconite bacterial communities. (**B**) Genus-level composition (top 10 most abundant classified genera) of cryoconite bacterial communities.

Taxonomic annotation identified 49 bacterial phyla, 676 families, and 1038 genera. Proteobacteria (19.17–51.35%), Cyanobacteria (3.52–28.73%), and Bacteroidota (9.38–34.87%) were the dominant phyla, followed by Actinobacteriota (5.04–18.76%), Acidobacteriota (0.49–9.40%), and Firmicutes (0.07–3.63%) (Figure 3A). Collectively, these phyla accounted for 87% of the microbial communities. Notably, the relative abundance of Cyanobacteria in South America was significantly lower than that on other continents. At the genus level, a considerable proportion of sequences remained unclassified, particularly in samples from Asia and Europe (Figure S1). Glacial environments’ extremity causes gaps in reference sequences for glacier-specific genera in databases. Short-read sequencing fails to capture fine inter-genus variations, lowering taxonomic resolution. Unclassified sequences reveal unexplored glacial microbial diversity but challenge genus-level analysis accuracy and ecological interpretations. Among the classifiable bacterial genera, *Phormidesmis_ANT.L52.6* (6.55%), *Ferruginibacter* (5.88%), and *Polaromonas* (4.31%) were the most dominant (Figure 3B). The predominant bacterial genera varied by continent: *Phormidesmis_ANT.L52.6* (15.22%) and *Ferruginibacter* (12.6%) in Antarctica; *Tychonema_CCAP_1459-11B* (5.65%) and *Polaromonas* (5.48%) in Asia; *Ferruginibiber* (9.11%) and *Hymenobacter* (4.70%) in Europe; *Phormidesmis_ANT.L52.6* (14.37%) and *Granulicella* (8.85%) in North America; and *Hymenobacter* (11.25%) and *Polaromonas* (10.24%) in South America.

**Figure 4:**
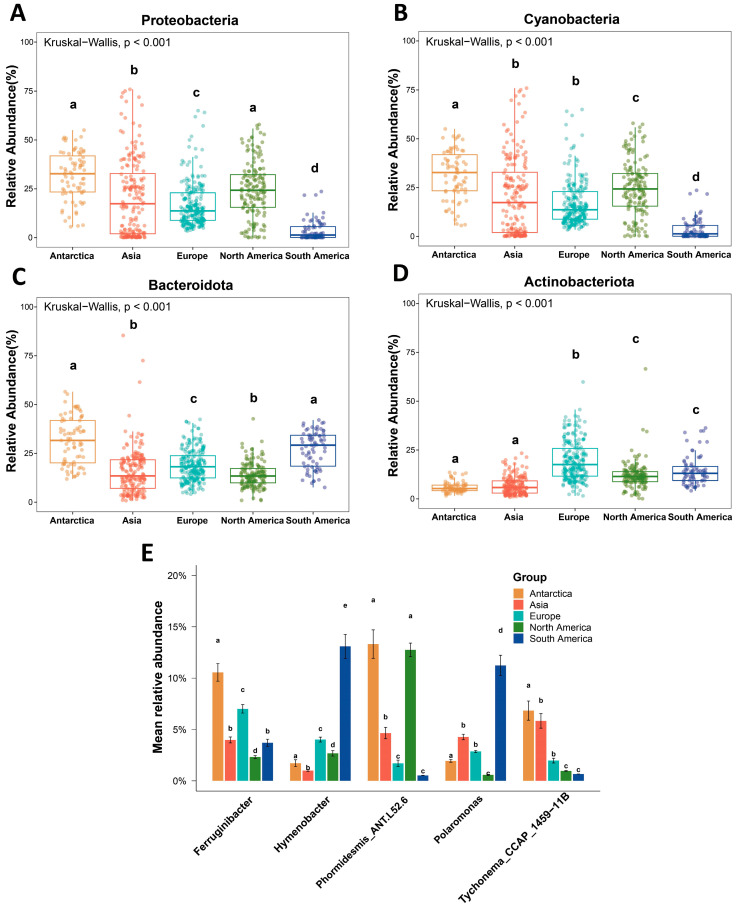
Analysis of differences in dominant species within cryoconite bacterial communities. (**A**–**D**) Differences in relative abundances of dominant bacterial phyla among the five continents. Bars sharing the same letter are not significantly different. (**E**) Differences in relative abundances of dominant bacterial genera among the five continents.

Kruskal–Wallis tests performed on the dominant phyla and genera revealed significant differences in their relative abundances across the different regions (*p* < 0.001) (Tables S5 and S6). The relative abundance distributions of major phyla, including Proteobacteria, Cyanobacteria, Actinobacteriota, and Bacteroidota, exhibited distinct geographic patterns (Figure 4A–D). Proteobacteria showed higher relative abundances in Asia and South America, whereas Bacteroidota was predominant in Antarctica. There are also significant differences in the relative abundance of dominant genera across regions (Figure 4E). *Ferruginibacter* shows relatively high abundance in Antarctica and Europe, while *Hymenobacter* and *Polaromonas* are mainly found in South America. Both *Phormidesmis_ANT.L52.6* and *Tychonema_CCAP_1459-11B*, belonging to the Cyanobacteria phylum, exhibit the highest relative abundance in Antarctica, followed by Asia and North America, with minimal abundance in South America, which is consistent with the inter-group differences in the relative abundance of the Cyanobacteria phylum. The variations in cryoconite bacterial communities at the dominant phylum and genus levels may stem from the influence of unique environmental conditions in different geographical regions, reflecting the regional distribution patterns of bacterial community structure.

### 3.3. Beta Diversity of Cryoconite Bacterial Communities

**Figure 5:**
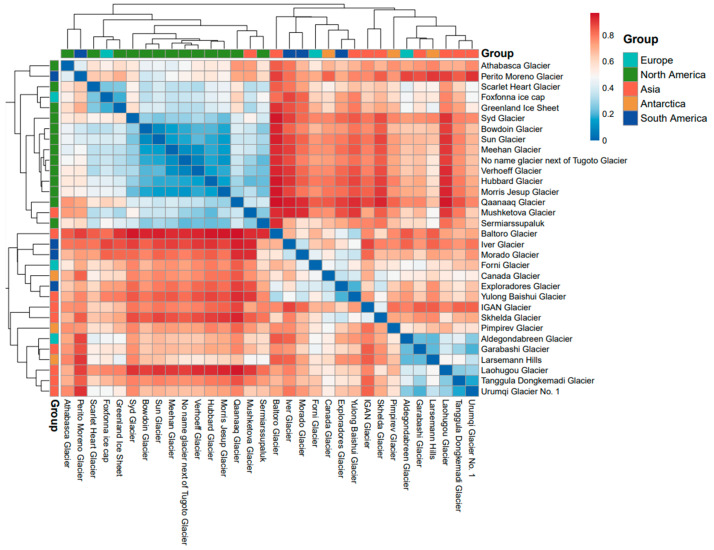
Heatmap of the Bray–Curtis distance matrix for cryoconite bacterial communities.

**Figure 6:**
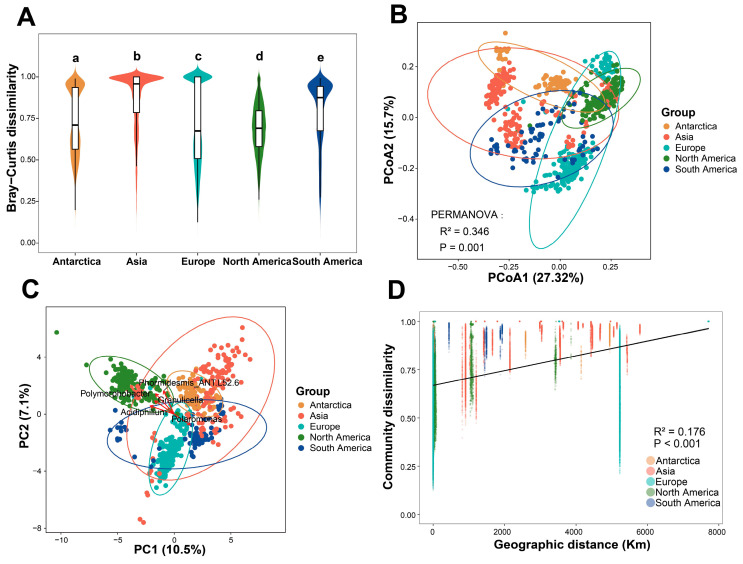
Beta diversity of cryoconite bacterial communities. (**A**) Differences in bacterial community beta diversity (based on Bray–Curtis dissimilarity) among continents. Groups with the same letter in the figure indicate no significant difference. (**B**) Principal Coordinates Analysis (PCoA) plot of cryoconite bacterial communities from the five continents based on the Sørensen distance. (**C**) Principal component analysis (PCA) depicts variation in the distribution of the top 5 Genera among continents. (**D**) The distance-decay relationship between bacterial community dissimilarity (based on Bray–Curtis distance) and geographic distance.

The heatmap of the Bray–Curtis dissimilarity matrix among glaciers reveals that North America exhibited notably high Bray–Curtis dissimilarity with other continents, indicating significant differences in bacterial communities (Figure 5). Compared to other groups, North America also demonstrated a more distinct clustering pattern. Beta diversity was assessed by calculating Bray–Curtis dissimilarities between samples grouped by continent (Figure 6A). Europe exhibited the lowest beta diversity, while North America showed the highest. The Kruskal–Wallis test revealed significant differences in bacterial community beta diversity (Bray–Curtis dissimilarity) among the continents (Table S7).

Principal Coordinates Analysis (PCoA) was conducted based on the Sørensen distance matrix to compare the bacterial community structures of ice dust from different continents (Figure 6B). The PCoA results revealed a distinct separation trend of bacterial communities across different continents in the multidimensional space. Furthermore, the Permutational Multivariate Analysis of Variance (PERMANOVA) results demonstrated that the differences in community composition among regions were statistically significant (R^2^ = 0.346, *p* = 0.001). At the genus level, the principal component analysis (PCA) revealed that 17.6% of the total variance was explained (PC1 = 10.5%, PC2 = 7.1%) (Figure 6C). Genera such as *Polymorphobacter*, *Phormidesmis_ANT.L52.6*, *Polaromonas*, *Acidiphilium*, and *Granulicella* scored highly on the PC1 and PC2 axes, making significant contributions to the differences among different continents. The distance-decay relationship was analyzed by examining the correlation between bacterial community similarity (Bray–Curtis dissimilarity) and geographical distance. Linear regression indicated a significant decline in community similarity with increasing geographical distance within continental regions (*p* < 0.001) (Figure 6D). The strength of this distance decay relationship varied substantially among regions, with the highest R^2^ value observed in South America (R^2^ = 0.4553) and the lowest in North America (R^2^ = 0.0132). The notably low R^2^ in North America primarily arises from the high spatial clustering of most samples, which renders the distance gradient ineffective, coupled with their location in Greenland, where extreme polar environmental heterogeneity is likely low, thereby undermining the capacity of geographic distance to effectively explain community similarity. This variation suggests that the explanatory power of geographical distance for bacterial community variation differs across continents. Overall, these results underscore geographical distance as a key driver shaping the biogeographic distribution of cryoconite bacterial communities, likely by limiting dispersal and exchange of bacterial taxa, thereby promoting regional differentiation in community composition.

### 3.4. Assembly Processes of Cryoconite Bacterial Communities

**Figure 7:**
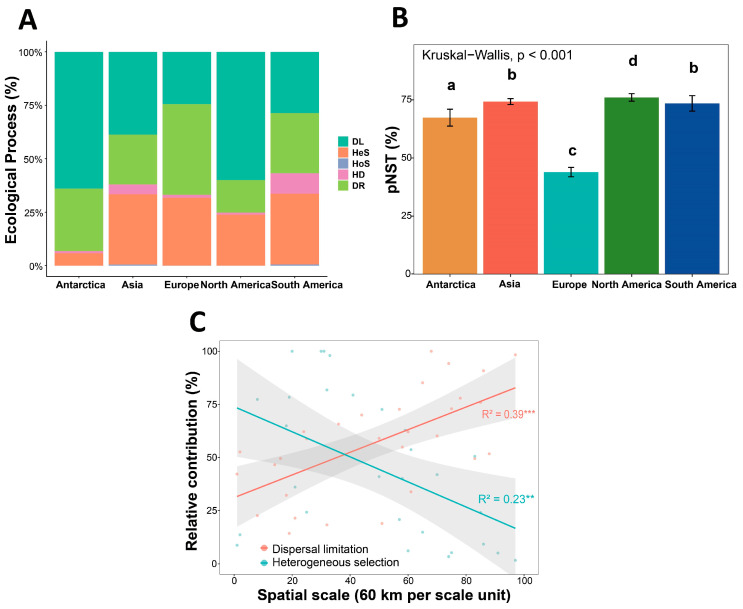
The assembly mechanisms and scale dependence underlying the diversity patterns of bacterial communities in cryoconite. (**A**) Null model analysis of cryoconite bacterial community assembly processes. DL: Dispersal limitation; HeS: Heterogeneous selection; HoS: Homogeneous selection; HD: Homogeneous dispersal; DR: Drift. HeS and HoS represent deterministic processes, while DR, DL, and HD represent stochastic processes. (**B**) Regional variation in phylogenetic normalized stochasticity ratio (pNST) of community assembly across continents. Groups with the same letter in the figure indicate no significant difference. (**C**) Evaluation of the relative contribution of bacterial community assembly processes with increasing spatial scales (60 km per scale unit) (**, *p* < 0.01; ***, *p* < 0.001). The grey shadow surrounding the regression line represents the 95% confidence interval.

Null model analysis was employed to quantify the relative contributions of different ecological processes to community assembly. The results indicated that dispersal limitation (DL) and heterogeneous selection (HeS) were the two dominant processes governing the spatial distribution of cryoconite bacterial communities (Figure 7A). Dispersal limitation (DL) accounted for a substantial proportion of community assembly across all regions, particularly in Antarctica and North America, highlighting the significant constraints imposed by geographical isolation and limited dispersal capacity. Concurrently, heterogeneous selection (HeS) also contributed considerably in Asia, Europe, and South America, reflecting the selective pressure of environmental heterogeneity on species composition in these regions. In contrast, homogeneous selection (HoS), homogenizing dispersal (HD), and drift (DR) generally exhibited lower contributions across the continents.

The phylogenetic null model was used to calculate the phylogenetic normalized stochasticity ratio (pNST), which revealed significant differences among regions (Figure 7B) (Table S8). Specifically, Asia and North America exhibited higher pNST values, indicating a greater influence of stochastic processes (e.g., dispersal limitation or drift) in their community assembly. Conversely, Europe demonstrated lower pNST values, suggesting that deterministic processes (e.g., environmental filtering or biotic interactions) exerted stronger effects in these regions.

As the spatial scale increased (with each unit representing 60 km), the relative contribution of dispersal limitation showed a significant positive trend (R^2^ = 0.39, *p* < 0.001), whereas the contribution of heterogeneous selection decreased significantly (R^2^ = 0.23, *p* < 0.01) (Figure 7C).

In summary, the assembly of cryoconite bacterial communities globally results from the combined effects of multiple ecological processes, whose relative importance varies regionally. The shifting dominance between deterministic (e.g., selection) and stochastic (e.g., dispersal limitation) processes across different geographic areas underscores the diversity and complexity of assembly mechanisms on a global scale. The neutral community model and null model analyses collectively demonstrate that the assembly of cryoconite bacterial communities is governed by both deterministic and stochastic processes, with stochasticity playing a predominant role.

### 3.5. Environmental Drivers of Cryoconite Bacterial Community Structure

**Figure 8:**
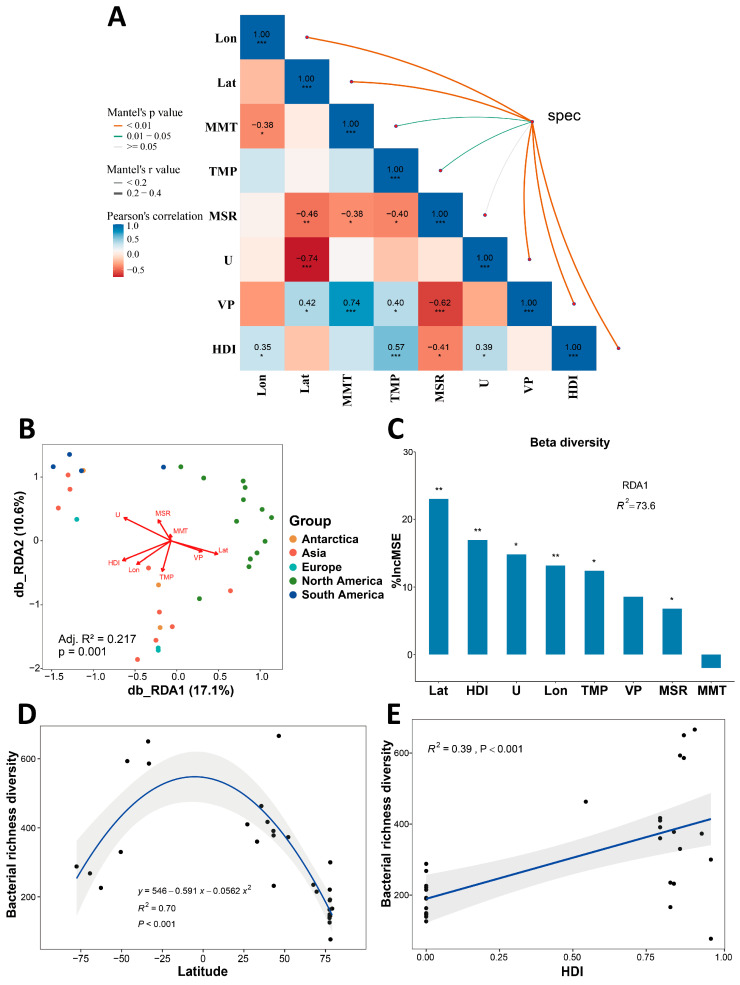
Dominant variables influencing the diversity of bacterial communities in cryoconite. (**A**) The connection between cryoconite bacterial communities and environmental drivers was visualized through Mantel test. Environmental drivers mainly include latitude (Lat), longitude (Lon), Global Human Development Index (HDI), wind speed (U), mean solar radiation (MSR), mean monthly temperature (MMT), total monthly precipitation (TMP), and water vapor pressure (VP). (**B**) Distance-based redundancy analysis (db-RDA) of the relationship between cryoconite bacterial communities and environmental factors. (**C**) Main variables affecting the beta diversity (based on Bray–Curtis distance) of the bacterial communities through random forest analysis. Feature importance ranked by %IncMSE. (*, *p* < 0.05; **, *p* < 0.01; ***, *p* < 0.001) (**D**) Quadratic relationships between the richness diversity of bacterial communities and Latitude. (**E**) Linear regression between cryoconite bacterial community richness and HDI.

To determine the relationships between geographic, anthropogenic, and climatic factors and cryoconite bacterial communities, Mantel tests were performed based on Bray–Curtis dissimilarity (Figure 8A). The results revealed significant correlations between species distribution and geographic distance, anthropogenic factors, and climate variables. Geographic factors exhibited the strongest influence on species composition, with both latitude (r = 0.266, *p* < 0.01) and longitude (r = 0.221, *p* < 0.01) showing strong correlations, indicating that spatial distance serves as a primary driver of community distribution by restricting species dispersal and establishing ecological gradients. Among anthropogenic factors, the Human Development Index (HDI) was strongly correlated with bacterial community structure (r = 0.263, *p* < 0.01). For climatic variables, wind speed (r = 0.259, *p* < 0.01) and vapor pressure (r = 0.241, *p* < 0.01) showed significant correlations with species composition, while mean monthly temperature (r = 0.163, *p* = 0.033) and precipitation (r = 0.185, *p* < 0.01) approached significance thresholds. Solar radiation (r = 0.119, *p* = 0.05) did not demonstrate a significant correlation. Distance-based redundancy analysis (db-RDA) further supported the significant role of environmental factors in driving cryoconite bacterial community distribution. The db-RDA showed that environmental variables explained 27.7% of the total variation in bacterial communities (RDA1 = 17.1%, RDA2 = 10.6%) (Figure 8B). The overall model fit was highly significant (Adjusted R^2^ = 0.2165, *p* < 0.001), indicating that the selected environmental factors could substantially explain the distributional variation in cryoconite bacterial communities.

Random Forest analysis was conducted with the RDA1 axis as the response variable and environmental factors as predictors (Figure 8C). Results showed that latitude, HDI, and wind speed had high %IncMSE values that reached statistical significance, indicating that model prediction accuracy decreased substantially when these variables were randomly permuted, thus confirming their key roles in driving bacterial community distribution. The Random Forest results were consistent with Mantel test findings, demonstrating the important influence of these environmental factors on bacterial beta diversity. The R^2^ value of 73.6% indicated that these environmental factors explained 72.9% of the variation in the RDA1 axis, highlighting their strong explanatory power for community distribution variation.

Random Forest analysis identified latitude and HDI as the most influential environmental factors affecting cryoconite bacterial community structure. To further investigate their relationships with bacterial diversity, we fitted models between these factors and diversity indices. Bacterial richness showed a nonlinear relationship with latitude (R^2^ = 0.7, *p* < 0.001), exhibiting a hump-shaped pattern with higher diversity at mid-latitudes and lower diversity in polar regions (Figure 8D). Bacterial richness was positively correlated with HDI (R^2^ = 0.39, *p* < 0.001) (Figure 8E).

Mantel tests and Random Forest analysis collectively revealed the association patterns between species composition and environmental factors: the assembly of the community is co-driven by geographical distance, anthropogenic influences, and climatic conditions, with the climatic gradient playing a lesser role in species turnover compared to geographical isolation and human factors.

## 4. Discussion

### 4.1. Latitude and HDI Drives the Geographic Distribution Patterns of Cryoconite Bacterial Communities

As a key component of polar and alpine ecosystems, cryoconite bacterial communities exhibit distinct distribution patterns in diversity and taxonomic composition at the global scale. In terms of alpha diversity, both the Richness and Shannon indices revealed significant differences among continents, with Antarctica showing relatively high diversity and South America exhibiting the lowest values, significantly different from other regions (Figure 2). Proteobacteria, Cyanobacteria, and Bacteroidota were the dominant phyla with high relative abundances, collectively constituting a major proportion of the microbial communities—a finding consistent with previous studies [61]. The proportions of these high-abundance taxa varied significantly across continents (Figure 4). Cyanobacteria, as crucial photoautotrophs in cryoconite, influence nutrient availability for the microbial community. Their highest abundance in Antarctica, where photosynthetic activity often exceeds respiration, facilitates the accumulation of organic carbon for heterotrophic organisms [62]. In contrast, the prevalence of mid-latitude and low-latitude alpine glaciers in South America correlates with minimal cyanobacterial abundance [63]. In these environments, the subversion of photosynthesis by respiration causes microbial communities to rely heavily on carbon subsidies from other ecosystems, creating impoverished and unstable nutrient pools (such as nitrogen and phosphorus) that may ultimately limit biodiversity [16]. Further analysis of beta diversity confirmed biogeographic distribution patterns, with clear clustering of bacterial communities by continent (Figure 6). Geographical distance and environmental heterogeneity play crucial roles in shaping microbial distribution [64]. Cryoconite environments, often situated in remote high-altitude or high-latitude regions, may impose dispersal barriers (e.g., mountains, oceans) that limit bacterial exchange over large spatial scales. Local physicochemical properties, such as pH, oxygen concentration, and organic matter content, also significantly influence bacterial community composition and diversity [65,66,67].

To investigate the drivers of cryoconite bacterial diversity and composition, this study integrated geographic, anthropogenic, and climatic factors. Due to data availability constraints, key physicochemical variables (e.g., pH, total organic carbon, total nitrogen) were not included, which may partially limit a comprehensive interpretation of local environmental filtering. Our analysis identified latitude and the Human Development Index (HDI) as key explanatory variables for cryoconite bacterial community variation (Figure 8). Latitude, often correlated with climatic gradients, reflects environmental heterogeneity across latitudinal zones. On the Tibetan Plateau, latitude was identified as the primary driver of bacterial beta diversity in the Dongkemadi Glacier [68], and global studies have confirmed its role in structuring glacial aquatic microbial diversity [66]. Cryoconite bacterial communities exhibited a unique latitudinal diversity pattern, with richness showing a hump-shaped relationship to latitude, increasing from polar to mid-latitudes. In typical terrestrial and marine ecosystems, microbial diversity often peaks around 35° latitude [69,70,71]. Although lower-latitude data were not included in this study, the observed increase in diversity from polar to mid-latitude regions suggests that the latitudinal diversity pattern of cryoconite bacterial communities aligns with the typical microbial latitudinal diversity distribution (Figure 8D). Community dissimilarity (beta diversity) increased with greater latitudinal differences, which often correspond to increased geographical distance, consistent with distance-decay relationships.

The significant positive correlation between the HDI and cryoconite bacterial community structure represents a relatively novel discovery (Figure 8E), indicating that human activities have extended beyond regional limits and are now involved in shaping global glacial ecosystems. This potential mechanism can be interpreted in two ways: First, regions with high HDI typically see more intensive industrial and agricultural activities, likely leading to increased atmospheric deposition of nutrients (e.g., nitrogen, phosphorus, organic carbon) that may alter the living environment of glacial microorganisms and relate to shifts in their community structure [72]; Second, frequent movements of people and goods (e.g., tourism, scientific expeditions, trade) may facilitate long-distance microbial dispersal, reducing the effects of geo-graphic isolation [73].

Future studies could incorporate more global cryoconite amplicon datasets and integrate high-resolution environmental parameters (e.g., physicochemical properties, organic nutrient data) to systematically quantify the intensity and mechanisms of environmental filtering, elucidate its interaction with geographic distance, and enhance predictive capabilities regarding microbial distribution patterns and ecological responses in extreme environments.

### 4.2. Scale-Dependent Effects on Cryoconite Bacterial Community Assembly Processes

This study revealed a significant increase in cryoconite bacterial community dissimilarity with increasing geographical distance, demonstrating a clear distance-decay relationship (Figure 6D). Stochastic processes, particularly dispersal limitation, played a major role in shaping bacterial community assembly at the continental scale. The contribution of dispersal limitation to community assembly progressively increased with geographical distance (Figure 7C), ultimately surpassing the influence of environmental selection as the dominant force—a finding consistent with studies on microbial biogeography in glacier-fed streams [74]. The shift in community assembly mechanisms from determinism-dominated at regional scales to stochasticity-dominated at continental scales highlights the scale dependency of ecological processes, which is crucial for understanding microbial biogeographic patterns.

At regional scales, environmental heterogeneity generates strong deterministic selection pressures, where microbial community structure is primarily governed by environmental filtering [75,76]. This selective process results in highly predictable community patterns. Environmental heterogeneity typically exerts a predominant influence on microbial community structure at smaller spatial scales. In contrast, the present study, spanning thousands of kilometers, provided a suitable spatial extent to investigate the effects of geographical distance [77]. When the study scope encompasses vast geographic barriers, microbial dispersal becomes severely constrained. Such long-distance dispersal limitations promote geographic isolation, thereby increasing stochasticity and spatial heterogeneity in community structure.

At the global scale, despite variations among glacial environments, they share core extreme characteristics such as low temperatures and high radiation. Under these conditions, the explanatory power of intercontinental environmental differences diminishes relative to the immense geographical distances. Whether a microorganism can reach a particular glacier (dispersal limitation) becomes more decisive for community composition than its adaptation to local conditions (environmental selection). Historical contingencies and stochastic dispersal events largely determine the species composition of extant microbial communities [78,79]. Additionally, some studies suggest that species’ niche breadths may expand at larger spatial scales, further attenuating the role of environmental filtering [80].

## 5. Conclusions

Through a systematic analysis of cryoconite bacterial communities across a transcontinental scale, this study elucidates their biogeographic distribution patterns and underlying assembly mechanisms. The results demonstrate significant differences in bacterial community composition across geographic locations. Geographic factors, represented by latitude, and anthropogenic influence, represented by the Human Development Index (HDI), collectively determined cryoconite bacterial community structure. Alpha diversity exhibited a hump-shaped relationship with latitude and was positively correlated with HDI. Regarding assembly mechanisms, the relative importance of deterministic and stochastic processes showed pronounced scale dependency: as the spatial scale expanded, community assembly shifted from environmentally driven selection at local scales to dispersal limitation-dominated processes at regional and continental scales. Notably, the positive influence of HDI on community composition appears to reveal the potentially profound impacts of socioeconomic activities on pristine glacier ecosystems, suggesting that human activities may have transcended geographical boundaries and hold the potential to become a significant driving force in the evolution of global glacier ecosystems. However, we also need to approach this result with caution. The HDI value of 0 in the Antarctic region may skew the overall data, making the link between HDI and bacterial richness seem more pronounced in current analyses. Thus, the stability and generality of HDI’s impact need further verification. Understanding such multi-scale, multi-factor (natural and anthropogenic) interactions is crucial for accurately predicting microbial community succession and shifts in ecological functions against the backdrop of ongoing glacier retreat.

## Data Availability

The sequences utilized in this study are publicly available and were previously published. They were obtained from the NCBI Sequence Read Archive. All accession numbers are provided in Supplementary Materials.

## Supplementary Materials

The following supporting information can be downloaded at: https://www.mdpi.com/article/10.3390/microorganisms14010162/s1, Figure S1: Genus composition of bacterial communities in cryoconite across different continents; Figure S2: The relationship between community similarity and geographical distance across five continents; Table S1. Detailed information on the 695 collected cryoconite samples, including the accession numbers, associated glaciers, country, continent, collection date, bioproject accession, longitude and latitude coordinates, sequencing primer pairs, sequencing technology, and DOI numbers; Table S2. Environmental variable data collected at the glacier scale, including latitude (Lat), longitude (Lon), wind speed (U), mean solar radiation (MSR), mean monthly temperature (MMT), total monthly precipitation (TMP), and water vapor pressure (VP), and HDI (Human Development Index). Among these, the climatic variable data represent the monthly averages for the sampling month of the samples, while the HDI is extracted based on the country where the glacier is located; Table S3: Significance of cryoconite bacterial richness and Shannon diversity in different continents, with *p*-values from Kruskal–Wallis and Dunn tests; Table S4. Community dissimilarity test based on ANOSIM using Bray–Curtis distance; Table S5. Significance of dominant bacterial phyla in cryoconite communities across different continents, with *p*-values from Kruskal–Wallis and Dunn tests; Table S6. Significance of dominant bacterial genera in cryoconite communities across different continents, with *p*-values from Kruskal–Wallis and Dunn tests; Table S7. Beta diversity differences (Bray–Curtis) across continents were assessed, with *p*-values from Kruskal–Wallis and Dunn tests; Table S8. Differences in pNST across continents were assessed, with *p*-values from Kruskal–Wallis and Dunn tests.
