# Supplementary material for: Biogeography of Cryoconite Bacterial Communities Across Continents
Source: Microorganisms. 2026 Jan 11;14(1):162. doi: 10.3390/microorganisms14010162 (PMC12844174; doi:10.3390/microorganisms14010162)
Supplement: Supplementary file 1 [file microorganisms-14-00162-s001.zip › supplementary materials.pdf]

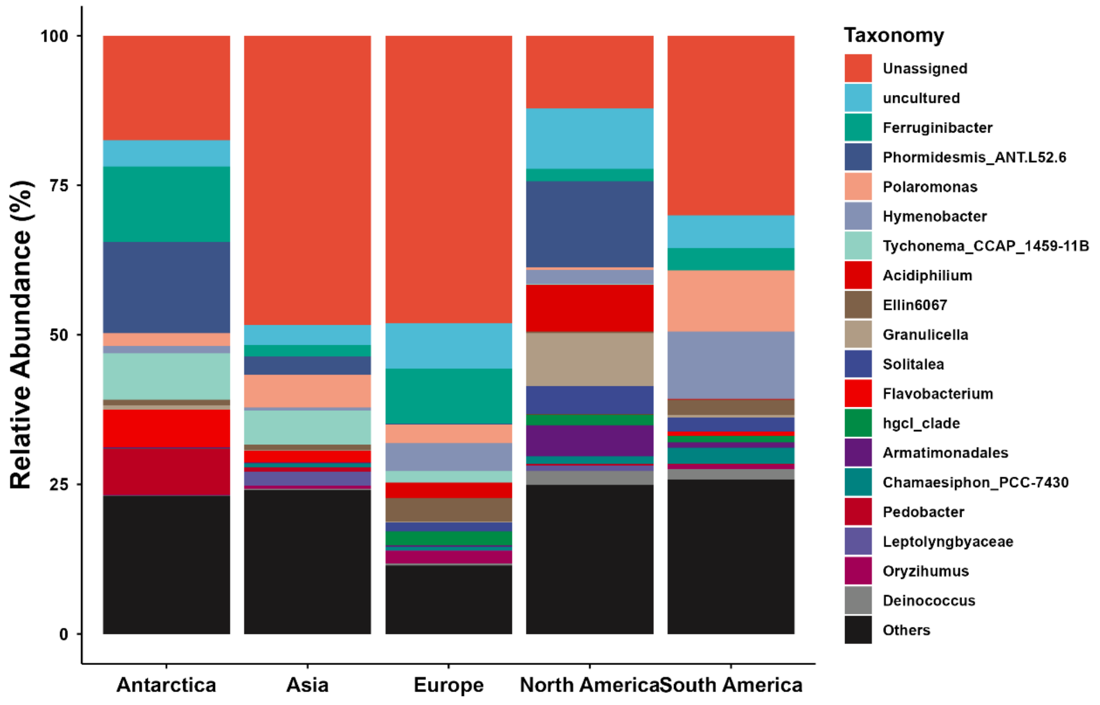

Figure S1. Genus composition of bacterial communities in cryoconite across different continents.

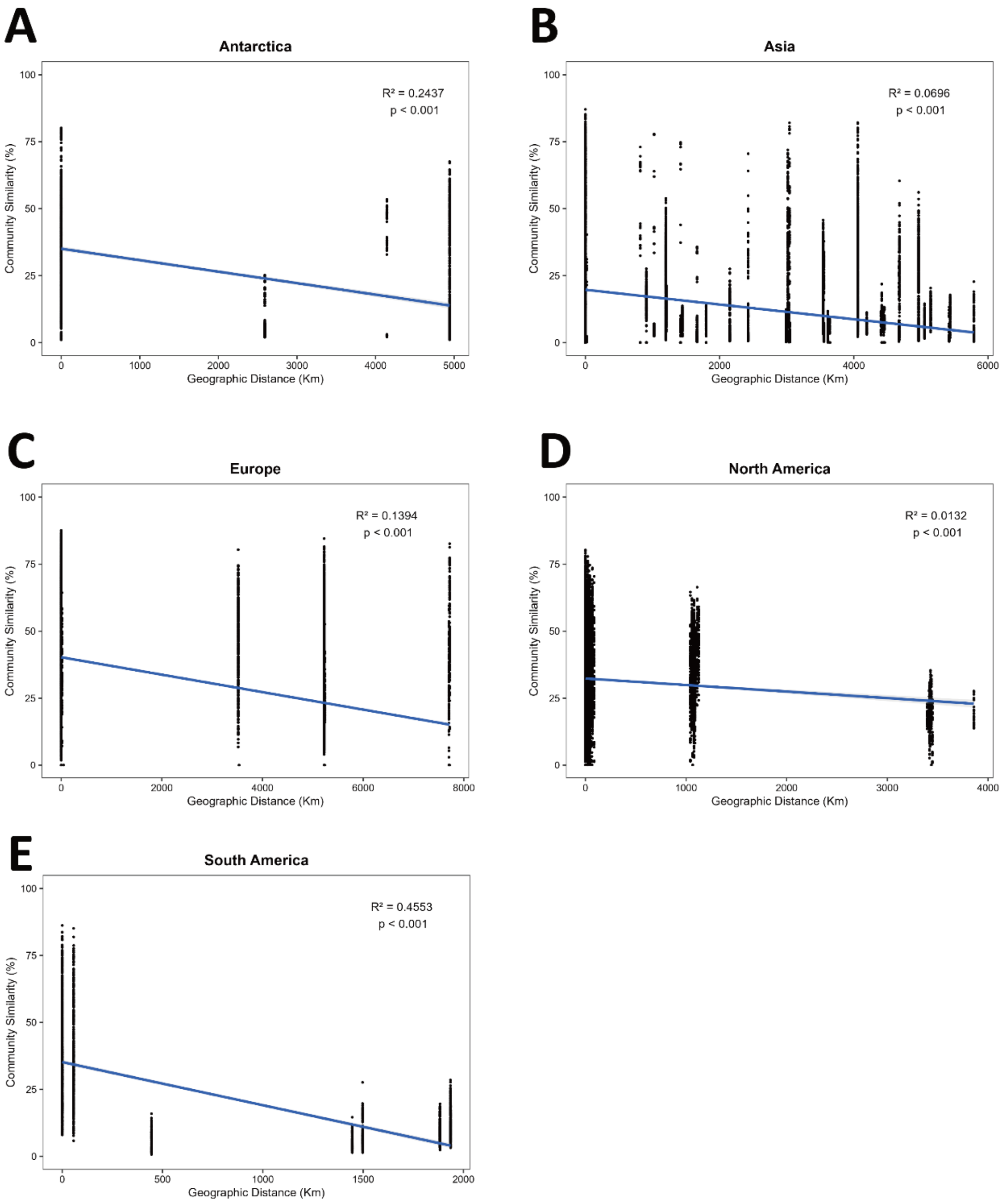

Figure S2. The relationship between community similarity and geographical distance across five continents.

Table S2. Environmental variable data collected at the glacier scale, including latitude (Lat), longitude (Lon), wind speed (U), mean solar radiation (MSR), mean monthly temperature (MMT), total monthly precipitation (TMP), and water vapor pressure (VP), and HDI (Human Development Index). Among these, the climatic variable data represent the monthly averages for the sampling month of the samples, while the HDI is extracted based on the country where the glacier is located.

Glacier	Continent	Lon(°)	Lat (°)	MMT (°C)	TMP(mm)	MSR($kJ \cdot m^{-2} \cdot day^{-1}$)	U($m \cdot s^{-1}$)	VP(kPa)	HDI
Canada Glacier	Antarctica	162.97	-77.62	-4.23	7.63	36168.09	3.80	0.21	0.00
Larsemann Hills	Antarctica	76.22	-69.42	-5.43	0.00	24908.32	6.20	0.28	0.00
Pimpirev Glacier	Antarctica	-60.41	-62.69	0.08	21.61	17829.49	4.90	0.57	0.00
Baltoro Glacier	Asia	76.65	35.70	-5.05	9.05	22321.08	4.87	0.13	0.54
Garabashi Glacier	Asia	42.46	43.31	0.25	93.80	15789.56	4.10	0.40	0.84
IGAN Glacier	Asia	66.03	67.58	6.10	83.15	10560.74	4.20	0.81	0.83
Laohugou Glacier	Asia	96.54	39.48	4.65	52.65	19522.61	3.93	0.47	0.80
Mushketova Glacier	Asia	101.86	79.10	-2.08	32.00	8728.57	4.75	0.50	0.83
Skhelda Glacier	Asia	42.65	43.19	6.39	91.60	15811.23	2.46	0.65	0.84
Tanggula Dongkemadi Glacier	Asia	92.08	33.07	2.12	102.42	18216.59	3.47	0.53	0.80
Urumqi Glacier No. 1	Asia	86.80	43.10	2.60	72.25	18640.00	4.20	0.57	0.80
Yulong Baishui Glacier	Asia	100.19	27.10	5.08	142.94	14448.45	3.29	0.85	0.80
Aldegondabreen Glacier	Europe	14.09	77.97	2.83	37.55	15063.90	3.91	0.69	0.97
Forni Glacier	Europe	10.59	46.40	4.15	59.78	17444.22	3.29	0.66	0.92
Foxfonna ice cap	Europe	16.07	78.08	3.40	35.39	9528.16	3.84	0.70	0.97
Athabasca Glacier	North America	-117.25	52.19	6.59	101.86	21425.67	2.56	0.62	0.94
Bowdoin Glacier	North America	-68.58	77.67	3.10	22.00	18342.55	2.70	0.66	0.00
Greenland Ice Sheet	North America	-49.81	69.58	-0.70	18.00	21508.54	2.10	0.52	0.00
Hubbard Glacier	North America	-67.82	77.54	5.41	23.00	18270.14	2.70	0.64	0.00
Meehan Glacier	North America	-70.31	77.87	4.79	24.00	18369.67	3.40	0.66	0.00
Morris Jesup Glacier	North America	-71.14	77.89	3.13	24.00	18421.91	3.70	0.67	0.00
No name glacier next of Tugoto Glacier	North America	-68.70	77.56	2.32	24.06	18236.01	2.88	0.62	0.00
Qaanaaq Glacier	North America	-69.15	77.50	-0.27	26.90	18138.93	3.28	0.60	0.00
Scarlet Heart Glacier	North America	-69.45	77.67	4.16	23.00	18327.12	2.90	0.66	0.00
Sermiarssupaluk	North America	-68.76	77.50	2.65	22.00	18352.97	2.70	0.66	0.00
Sun Glacier	North America	-69.46	77.78	3.55	25.07	18255.13	3.17	0.62	0.00
Syd Glacier	North America	-68.70	77.56	2.32	24.06	18236.01	2.88	0.62	0.00
Verhoeff Glacier	North America	-69.87	77.86	2.20	23.57	18359.90	3.06	0.66	0.00

Exploradores Glacier	South America	-70.18	-46.52	17.20	15.00	18343.42	7.40	0.82	0.87
Iver Glacier	South America	-70.23	-33.25	-0.81	10.18	20632.41	7.44	0.22	0.88
Morado Glacier	South America	-70.06	-33.75	4.92	9.09	21389.64	5.79	0.39	0.88
Perito Moreno Glacier	South America	-73.12	-50.51	8.87	82.82	12829.27	7.67	0.73	0.87

Table S3. Significance of cryoconite bacterial richness and shannon diversity in different continents, with p-values from Kruskal-Wallis and Dunn tests.

Group	p-value for Richness	p-value for Shannon
Antarctica - Asia	0.147	0.193
Antarctica - Europe	0.2	0.079
Asia - Europe	0.733	0.541
Antarctica - North America	< 0.001	< 0.001
Asia - North America	< 0.001	< 0.001
Europe - North America	< 0.001	< 0.001
Antarctica - South America	< 0.001	< 0.001
Asia - South America	< 0.001	< 0.001
Europe - South America	< 0.001	< 0.001
North America - South America	0.006	0.279

Table S4. Community dissimilarity test based on ANOISM using Bray-Curtis distance.

	r	P	Permutations
Antarctica- Asia	0.1637	0.001	999
Antarctica- Europe	0.5410	0.001	999
Antarctica- North America	0.7988	0.001	999
Antarctica- South America	0.4472	0.001	999
Asia- Europe	0.4489	0.001	999
Asia- North America	0.5800	0.001	999
Asia- South America	0.4063	0.001	999
Europe- North America	0.5412	0.001	999
Europe- South America	0.4781	0.001	999
North America- South America	0.7804	0.001	999
Global	0.4948	0.001	999

Table S5. Significance of dominant bacterial phyla in cryoconite communities across different continents, with p-values from Kruskal-Wallis and Dunn tests.

Group	p-value			
	Proteobacteria	Cyanobacteria	Bacteroidota	Actinobacteriota
Antarctica - Asia	< 0.001	< 0.001	< 0.001	0.264
Antarctica - Europe	< 0.001	< 0.001	< 0.001	< 0.001
Asia - Europe	< 0.001	0.624	< 0.001	< 0.001
Antarctica - North America	0.332	0.003	< 0.001	< 0.001
Asia - North America	< 0.001	< 0.001	0.229	< 0.001
Europe - North America	< 0.001	< 0.001	< 0.001	< 0.001
Antarctica - South America	< 0.001	< 0.001	0.219	< 0.001
Asia - South America	< 0.001	< 0.001	< 0.001	< 0.001
Europe - South America	0.004	< 0.001	< 0.001	0.003
North America - South America	< 0.001	< 0.001	< 0.001	0.063

Table S6. Significance of dominant bacterial genera in cryoconite communities across different continents, with p-values from Kruskal-Wallis and Dunn tests.

Group	p-value				
	Ferruginibacter	Hymenobacter	Phormidesmis_ANT.L52.6	Polaromonas	Tychonema_CCAP_1459-11B
Antarctica-Asia	< 0.001	0.047	< 0.001	0.001	0.003
Antarctica-Europe	0.018	< 0.001	< 0.001	0.003	< 0.001
Antarctica-North America	< 0.001	< 0.001	0.733	< 0.001	< 0.001
Antarctica-South America	< 0.001	< 0.001	< 0.001	< 0.001	< 0.001
Asia-Europe	< 0.001	< 0.001	< 0.001	0.436	0.234
Asia-North America	0.009	< 0.001	< 0.001	< 0.001	< 0.001
Asia-South America	0.752	< 0.001	< 0.001	< 0.001	< 0.001
Europe-North America	< 0.001	< 0.001	< 0.001	< 0.001	< 0.001
Europe-South America	< 0.001	< 0.001	0.177	< 0.001	< 0.001
North America-South America	0.018	< 0.001	< 0.001	< 0.001	0.293

Table S7. Beta diversity differences (Bray-Curtis) across continents were assessed, with p-values from Kruskal-Wallis and Dunn tests.

Group	p-value
Antarctica - Asia	< 0.001
Antarctica - Europe	< 0.001
Asia - Europe	< 0.001
Antarctica - North America	< 0.001
Asia - North America	< 0.001
Europe - North America	< 0.001
Antarctica - South America	< 0.001
Asia - South America	< 0.001
Europe - South America	< 0.001
North America - South America	< 0.001

Table S8. Differences in pNST across continents were assessed, with p-values from Kruskal-Wallis and Dunn tests.

Group	p-value
Antarctica - Asia	< 0.001
Antarctica - Europe	< 0.001
Asia - Europe	< 0.001
Antarctica - North America	< 0.001
Asia - North America	< 0.001
Europe - North America	< 0.001
Antarctica - South America	< 0.001
Asia - South America	0.152
Europe - South America	< 0.001
North America - South America	< 0.001
